# Discrepancies in Adolescent–Parent Perceptions of Parental Phubbing and Their Relevance to Adolescent Smartphone Dependence: The Mediating Role of Parent–Child Relationship

**DOI:** 10.3390/bs13110888

**Published:** 2023-10-27

**Authors:** Shi Chen, Dongqing Qiu, Xing Li, Qingbai Zhao

**Affiliations:** 1 School of Medical Humanities, Hubei University of Chinese Medicine, Wuhan 430065, China; 2 Key Laboratory of Adolescent Cyberpsychology and Behavior (CCNU), Ministry of Education, Wuhan 430079, China; 3 Key Laboratory of Human Development and Mental Health of Hubei Province, School of Psychology, Central China Normal University, Wuhan 430079, China; 4 School of Psychology, Central China Normal University, Wuhan 430079, China; 5 Jingzhou Vocational College of Technology, Jingzhou 434000, China

**Keywords:** parent–adolescent perceptual discrepancies in parental phubbing, parent–child relationship, smartphone dependence

## Abstract

Parental phubbing behavior is a significant predictor of adolescent smartphone dependence. However, previous research has mainly focused on the child and adolescent’s perspective, overlooking potential differences in how parents and their children perceive parental phubbing. Therefore, this study investigates whether disparities exist in how parents and adolescents perceive parental phubbing and how these perceptual differences impact adolescent smartphone dependence. We also explore the role of the parent–child relationship in this context. In this study, 728 families from a middle school in Wuhan were selected and surveys were administered to both children and parents. The findings are as follows: (1) Significant perceptual differences were found between parents and adolescents regarding parental phubbing. (2) These perceptual discrepancies positively predict adolescent smartphone dependence and negatively impact parent–child relationships. Additionally, parent–child relationships have a negative influence on adolescent smartphone dependence. (3) The parent–child relationship serves as a mediator between perceptual differences in parental phubbing behavior and adolescent smartphone dependence. In summary, this research highlights the importance of considering both parent and adolescent perspectives on parental phubbing and emphasizes the role of the parent–child relationship in influencing adolescent smartphone dependence.

## 1. Introduction

Smartphone dependency refers to excessive smartphone usage where individuals struggle to control their behavior, even in situations where phone use is prohibited, leading to adverse impacts on both their social and personal lives [1]. Adolescents, in contrast to adults, have heightened sensory needs, requiring more external stimuli to reach optimal arousal levels [2]. Smartphones, serving as multifaceted sources of leisure, entertainment and social connectivity, offer adolescents numerous opportunities for sensory gratification. Due to the incomplete development of self-regulatory capacities among adolescents, they are particularly vulnerable to smartphone dependency, resulting in unfavorable psychological outcomes such as reduced concentration, impaired memory and emotional issues [3,4]. Consequently, researchers have placed significant emphasis on understanding the challenges posed by adolescent smartphone usage.

Previous research has identified numerous factors influencing adolescent smartphone dependency, encompassing intrinsic device attributes, adolescent personality traits, family dynamics, peer relationships and environmental factors [5,6,7,8]. Notably, family factors have garnered significant attention, especially the pervasive issue of parental phubbing within the family context. Parental phubbing, defined as parents directing their attention toward smartphones during parent–child interactions, thereby neglecting their children [9], has been shown to jeopardize adolescents’ mental and physical well-being. In families where parental phubbing is more prevalent, adolescents are more likely to experience symptoms such as depression, poor sleep quality, academic procrastination, peer detachment and even suicidal tendencies [9,10,11,12,13]. Research indicates that parental phubbing positively predicts adolescent smartphone addiction, with factors like parent–child bonding, deviant peer associations and tendencies toward boredom mediating these effects [9,14,15]. Notably, Wang and colleagues found that parental phubbing not only impacts adolescent smartphone dependence but also extends its influence to academic performance [16]. Drawing from the parental acceptance–rejection (PAR) theory [17], early experiences of perceived parental acceptance or rejection significantly shape a child’s emotional, behavioral and socio-cognitive development [18]. These effects persist into adolescence and adulthood. Parental phubbing leaves adolescents feeling neglected in terms of positive attention and emotional feedback, prompting them to seek emotional gratification through smartphones. Adolescents who have experienced parental rejection may be more vulnerable to developing psychological issues [19,20], including smartphone addiction.

In previous research, the assessment of parental phubbing has primarily relied on the child’s perspective, with limited attention given to exploring how parents perceive their own engagement in such behavior. As a result, does a perceptual discrepancy exist between parents and their children regarding parental phubbing behavior? In the field of developmental psychopathology research, examining family or parental contributions to child psychopathology often involves gathering reports from multiple informants. These informants typically include children, parents, teachers, peers and clinical professionals, and their reports cover various aspects such as academic performance and behavioral records. However, discrepancies in these reports are not uncommon and can manifest as perceptual discrepancies, where different individuals hold contrasting subjective perceptions of a shared objective reality [21,22,23,24]. The attribution bias context model offers insight into this phenomenon by suggesting that incongruent attributions for the same behavior by the actor and the recipient create a perceptual gap between them. Particularly in cases where an individual’s actions lead to unfavorable outcomes within an interactive context, the actor tends to attribute these outcomes to external or interpersonal factors, while the recipient attributes them to the actor’s inherent characteristics [23]. Building on this framework, we propose Hypothesis 1: there may be perceptual discrepancies regarding parental phubbing between parents and their children.

If there is a perceptual gap between parents and adolescents concerning parental phubbing behavior, could this difference in perception become another influencing factor in adolescent smartphone dependency? Unlike the parental acceptance–rejection (PAR) theory, which primarily focuses on the one-way impact of parental behaviors on children, the “Discrepancy-Maladaptive” hypothesis proposed by De Los Reyes and Ohannessian explains this from the perspective of perceptual disparities between parents and children regarding the same behavior [25]. This theoretical framework suggests that the “discrepancy” observed in the parent–child dynamic indicates parents’ limited insight into their offspring’s actual circumstances, leading to an inadequate understanding of the child’s subjective perceptions. Within such a framework, adolescents become more prone to adverse developmental outcomes, which typically span behavioral, emotional, social adaptive and academic domains [21,23,25]. Presently, studies grounded in this hypothesis mainly focus on perceived disparities in parenting styles. Positive parenting behaviors are typically less acknowledged by adolescents than by their parents, while negative parenting behaviors are often perceived in excess by adolescents compared to parental recognition. These dynamics correlate with an increased susceptibility to negative developmental outcomes, including externalizing behaviors, emotional disturbances, social adaptation challenges and compromised academic achievements [21,26,27,28]. A meta-analysis suggests that the predictive power of parent–child perceptual discrepancies on developmental outcomes may exceed the impact of parenting styles reported solely by adolescents [29]. In parallel with these concepts, parental phubbing can be seen as a form of negative parental behavior and adolescent smartphone dependence as a manifestation of externalizing problem behavior. Parents’ phubbing behavior makes children more susceptible to smartphone dependence. In this context, we propose Hypothesis 2: perceptual discrepancies regarding parental phubbing positively predict adolescent smartphone dependence.

Furthermore, if the perceptual disparities regarding parental phubbing indeed turn out to be an influencing factor in adolescent smartphone dependence, how does this influence manifest? Within the family environment, the parent–child relationship holds a central position. The dynamics of interaction and emotional connection between parents and their children have a profound impact on the prospective social interactions of adolescents. According to attachment theory, establishing secure attachment bonds with parents provides adolescents with a foundation of nurturing support [30]. Conversely, the failure to foster such secure attachment bonds increases adolescents’ vulnerability to psychological and behavioral issues, making them more prone to the clutches of smartphone addiction [31]. Qualitative interviews have revealed that many full-time mothers cite their use of electronic devices at home as a means of temporarily escaping the monotony and frustrations of parenting, seeking respite from the burdens of domestic minutiae [32]. Building on these insights, parental phubbing is considered a manifestation of negative parenting behavior, encompassing practices such as harsh discipline, neglect, dishonesty and disregard. Drawing from the “Discrepancy-Maladaptive” hypothesis, when there is a perceptual discrepancy between parents and adolescents regarding parental phubbing behavior, adolescents may become dissatisfied with their parents. This dissatisfaction could potentially lead to an avoidance attachment strategy, resulting in lower communication quality, diminished trust and, ultimately, a decline in the quality of the parent–child attachment bond. Relevant research also confirms that divergent perceptions of parental behaviors between adolescents and parents may be associated with a poorer quality relationship between them [33]. Hence, we propose Hypothesis 3: perceptual discrepancies regarding parental phubbing significantly negatively predict the quality of the parent–child relationship. Additionally, a substantial body of research has established that parent–child relationships significantly predict adolescent smartphone dependence. Adolescents in harmonious parent–child relationships exhibit lower levels of smartphone dependence [31,34]. In summary, we put forward Hypothesis 4: the parent–child relationship acts as a mediator between perceptual discrepancies regarding parental phubbing and adolescent smartphone dependence.

**Figure 1:**
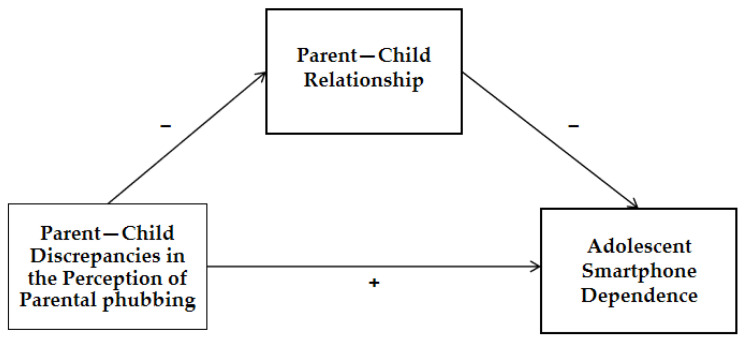
Theoretical framework of the mediation model.

The present study endeavors to examine the influence of perceptual disparities concerning parental phubbing on adolescent smartphone dependence, with a subsequent exploration of the mediating role of the parent–child relationship. The theoretical framework, illustrated in Figure 1, outlines the mediation model. Among the diverse landscape of adolescent demographics, middle school students represent a unique cohort characterized by the coexistence of emerging adult-like cognitions and lingering immaturity. This duality fosters a potent sense of independence, often coupled with emotional ambivalence in their interactions with adults. Consequently, middle school students are increasingly drawn to the digital realm while maintaining a significant reliance on parental figures for understanding, support and protection. Thus, this study places particular emphasis on the middle school student demographic.

## 2. Methods

### 2.1. Participants

This study focused on first-year and second-year middle school students and their parents from a middle school in Wuhan, China. A total of 1521 questionnaires were distributed and, after eliminating inadequately completed responses (such as those with identical responses for all items or completion times below 60 s), 1456 valid questionnaires were collected. The paired parent–student samples accounted for 728 responses, with 366 from female students, resulting in an effective response rate of 95.73%. Ethical approval for this study was obtained from the Ethical Committee for Scientific Research at the researchers’ affiliated institution and it adhered to ethical guidelines for the protection of human participants. All participants voluntarily took part in the experiment, provided written informed consent and received a small gift as an incentive.

### 2.2. Measures

#### 2.2.1. Predictive Variables

Perceptual Discrepancies in Parental Phubbing. To assess perceptual discrepancies in parental phubbing, students completed the parental phubbing scale [35], which consisted of 9 items (Cronbach’s α = 0.87) scored on a 5-point scale. Participants selected an option that best described their situation based on item descriptions (e.g., “When I eat with my parents, they use their phones”). Response options ranged from “1” (strongly disagree) to “5” (strongly agree), with higher scores indicating a higher level of parental phubbing. The parent’s questionnaire was adapted from the student’s version with subject replacement (e.g., “When I eat with my child, I use my phone”), also comprising 9 items (Cronbach’s α = 0.83).

Perceived discrepancies were computed using the difference score method. This involved standardizing scores separately reported by parents and students. The perceptual discrepancies were represented by the student’s Z-score minus the parent’s Z-score, which allowed for accounting for the influence of distribution disparities in original scores and equalizing the contribution of scores from different reporters [22,29].

#### 2.2.2. Outcome Variable

Adolescent Smartphone Dependence. To assess adolescent smartphone dependence, the smartphone addiction scale [36] was employed, comprising 32 items (internal consistency reliability was 0.95). Adolescents were presented with a series of statements (e.g., “I become impatient and restless when my smartphone is not within reach” and “I immediately check social media apps when I wake up”). This scale employed a 6-point scoring system, with higher scores indicating a more profound level of smartphone dependence among middle school students. The scale encompassed six dimensions: daily-life disturbance, positive anticipation, withdrawal, cyberspace-oriented relationship, overuse and tolerance.

#### 2.2.3. Mediating Variable

Parent–Child Relationship. The parent–child closeness scale, developed by Buchnan, Maccoby and Dornbush [37], was utilized in this study. It includes 2 subscales, one for father–child relationships (Cronbach’s α = 0.89) and the other for mother–child relationships (Cronbach’s α = 0.88), each consisting of 9 items (sample report item: “Do your parents show interest in talking to you when you want to speak?”). A 5-point rating scale was used, ranging from 1 for “completely disagree” to 5 for “completely agree”. The average score reflected the parent–child relationships, with higher scores indicating a closer relationship between adolescents and their parents.

### 2.3. Procedure

During the psychology course, adolescent questionnaires were distributed to students in each class and these questionnaires were completed by the students in the classroom setting. Initially, a test administrator provided participants with instructions, explained the assessment’s purpose, outlined response procedures and emphasized the principles of confidentiality. Following this introduction, adolescents proceeded to complete the questionnaires. Parental questionnaires were provided to students for subsequent completion by their respective parents.

### 2.4. Statistical Analyses

To rigorously assess potential common method bias, we conducted Harman’s single-factor test by loading all variables into an unrotated exploratory factor analysis [38]. The outcomes of this assessment revealed a presence of 19 factors with eigenvalues surpassing the critical value of 1. However, the initial factor only accounted for a cumulative variance of 12.32%, distinctly below the critical threshold of 40%. These results suggest that common method bias is not a major concern in this study.

The statistical package SPSS 19.0 and its associated plugins were employed for an array of analytical operations encompassing descriptive statistics, correlation analysis, independent samples *t*-tests, mediation tests and response surface analysis.

Response surface analysis was conducted through a two-step process. Step one incorporated polynomial regression to integrate children’s perceived scores (*C*), parents’ perceived scores (*P*), children’s perceived squared scores (*C*^2^), the multiplicative interaction term of parents’ perceived scores and children’s perceived scores (*C* × *P*) and parents’ perceived squared scores (*P*^2^) into a polynomial regression model (as expressed below). If the interaction term’s predictive effect (coefficient *b*_4_) proved significant, subsequent simple slope analysis was employed to explore the impact of perceptual discrepancies on the outcome variable: *Y = b*_0_ *+ b*_1_*C + b*_2_*P + b*_3_*C*^2^ *+ b*_4_*C × P + b*_5_*P*^2^ *+ e*. Step two encompassed the computation of polynomial regression coefficients, yielding four response surface analysis coefficients: *a*_1_ *= b*_1_ *+ b*_2_, testing the linear predictive effect of parental and student perceptions being aligned, whether the smartphone dependence of middle school students changes linearly when parent–child perceptions of parental phubbing exhibit a similar trend. *a*_2_ *= b*_3_ *+ b*_4_ *+ b*_5_, testing the quadratic predictive effect of parental and student perceptions being aligned. *a*_3_ *= b*_1_ *− b*_2_, testing for the linear predictive effect of parent–child perceptual discrepancies (student–parent). *a*_4_ *= b*_3_ *− b*_4_ *+ b*_5_, assessing the quadratic predictive effect of parent–child perceptual discrepancies. Lastly, the SPSS plugin PROCESS was employed to investigate the mediating effect of parent–child relationships within the impact of parent–child perceptual discrepancies in parental phubbing on middle school students’ smartphone dependence [39].

## 3. Results

### 3.1. The Discrepancies in Adolescent–Parent Perceptions of the Parental Phubbing

**Figure 2:**
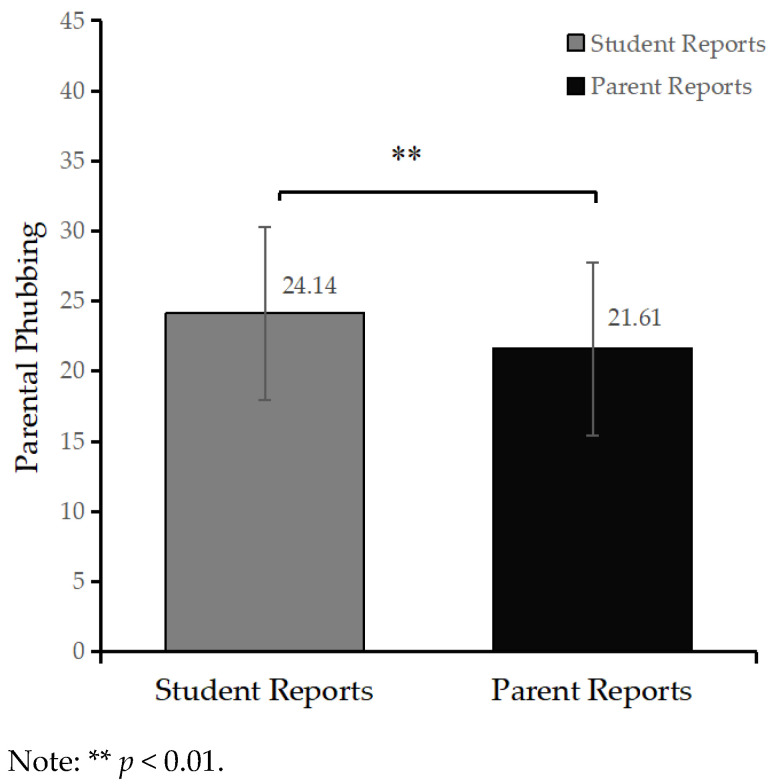
The perceived scores of parental phubbing behavior reported separately by students and their parents.

We conducted a paired-sample *t*-test to assess the differences between adolescent-reported and parental-reported measures of parental phubbing. The detailed results are presented in Figure 2. Notably, adolescent-reported scores (*M* = 24.14, *SD* = 7.59) were significantly higher than those reported by parents (*M* = 21.61, *SD* = 6.16), *t* (727) = 9.13, *p* < 0.01. This observed discrepancy demonstrated a moderate effect size (Cohen’s *d* = 0.34). These findings support our first hypothesis.

### 3.2. Correlation Analysis

The correlation analysis was conducted and the results are presented in Table 1. It is evident from the table that significant correlations exist between parent–child perceptual disparities in parental phubbing and both the parent–child relationship and middle school students’ smartphone dependence.

### 3.3. Predictive Effect of Parent–Child Perceptual Discrepancies in Parental Phubbing on Adolescent Smartphone Dependence

**Figure 3:**
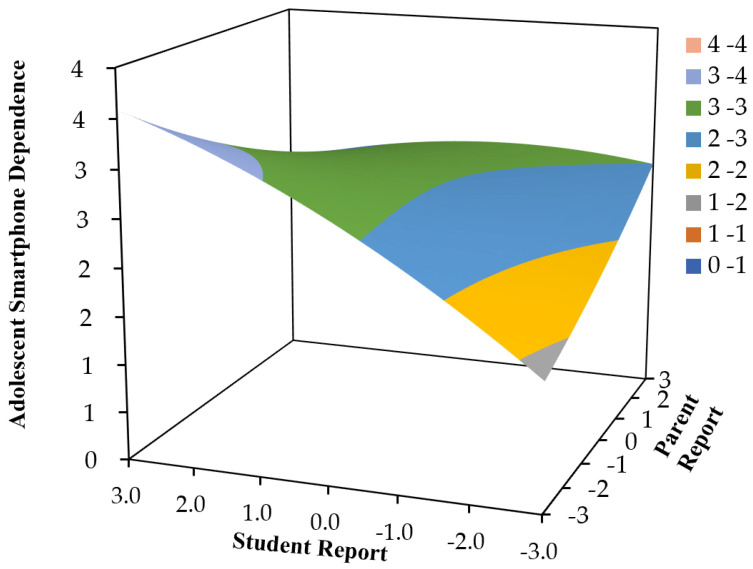
The response surface between students’ perception of parental phubbing (*X*), parents’ perception of their phubbing (*Y*) and students’ smartphone dependency (*Z*).

We constructed a comprehensive response surface analysis model, with student perceptions of parental phubbing on the *X*-axis, parental perceptions on the *Y*-axis and middle school students’ smartphone dependence on the *Z*-axis. Coefficients for the polynomial regression model and response surface analysis are presented in Table 2 and the visualized outcomes are depicted in Figure 3. In the relationship between parental phubbing perception and smartphone dependence in middle school students, a consistent positive linear effect was statistically significant (*a*_1_ = 0.15, *p* < 0.001). This indicates that as both parental and student perceptions of parental phubbing increased, middle school students’ smartphone dependency also escalated. Furthermore, a noteworthy positive linear effect of disparities (*a*_3_ = 0.18, *p* < 0.01) emphasized that greater parent–child perceptual discrepancies (student perceptions subtracted from parental perceptions) corresponded to heightened levels of middle school students’ smartphone dependency.

### 3.4. Mediation Analysis

In the mediation analysis, we employed parent–child perceptual discrepancies in parental phubbing as the independent variable, middle school students’ smartphone dependency as the dependent variable and the parent–child relationship as the mediating variable. Additionally, we controlled for student grade level. The mediation analysis was conducted using Model 4 of the SPSS plugin PROCESS [20].

**Figure 4:**
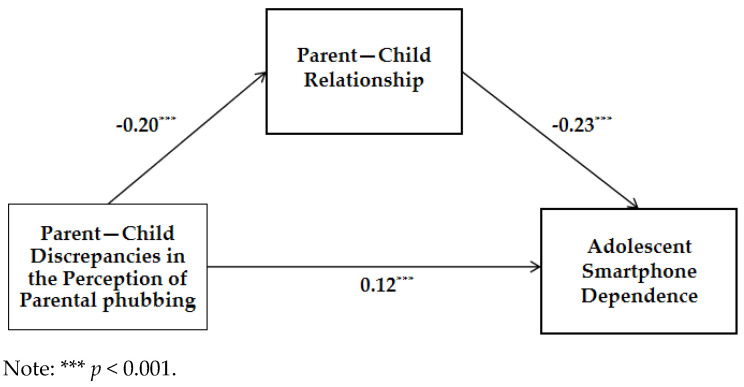
The result of the mediation model.

As presented in Table 3, the overall regression equation showed significance, *R*^2^ = 0.08, *F* (3724) = 19.833, *p* < 0.001. To validate the mediation effect, we utilized the Bootstrap resampling methodology and the results are depicted in Figure 4. The findings indicate that parent–child perceptual discrepancies in parental phubbing significantly and positively predicted middle school students’ smartphone dependency, ***β*** = 0.12, *p* < 0.01, 95%CI = [1.25, 5.06]. Furthermore, these discrepancies had a negative impact on the parent–child relationship, ***β*** = −0.20, *p* < 0.01, 95%CI = [−3.85, −1.8], and the parent–child relationship inversely influenced middle school students’ smartphone dependency, ***β*** = −0.23, *p* < 0.01, 95%CI = [−0.56, −0.28]. The overall effect size was 0.17 (*p* < 0.01), with a direct effect size of 0.12 (*p* < 0.01). The cumulative indirect effect stemming from the parent–child relationship amounted to 0.05 (95%CI = [0.03, 0.07]). Consequently, the mediating effect accounted for 27.05% of the total effect. These results provide validation for our hypotheses 2, 3 and 4.

## 4. Discussion

Grounded in the “Discrepancy-Maladaptive” hypothesis, this study explored the latent relationship between parent–child perceptual discrepancies in parental phubbing and adolescent smartphone dependence, unveiling the mediating role of the parent–child relationship. The outcomes of this study broaden the scope of the “Discrepancy-Maladaptive” hypothesis, potentially offering a theoretical perspective for future interventions targeting adolescent smartphone dependence.

Drawing upon the “Discrepancy-Maladaptive” hypothesis, this study delved into the underlying connection between parent–child perceptual disparities regarding parental phubbing and adolescent smartphone dependence. In doing so, it unveiled the mediating influence of the parent–child relationship. The findings of this study expand the horizons of the “Discrepancy-Maladaptive” hypothesis, potentially providing a valuable theoretical framework for future interventions aimed at mitigating adolescent smartphone dependence.

### 4.1. Perceptual Discrepancies in Parental Phubbing between Parents and Adolescents

The results highlight the presence of significant perceptual disparities in parental phubbing between parents and adolescents. Specifically, parents’ reported perceptions significantly lag behind those of adolescents, indicating that parents might be less aware of their excessive phubbing compared to their children. This finding resonates with previous research on perceptual disparities in parenting styles [28]. Drawing from the attribution bias context model [23], within interactive contexts, negative outcomes are often attributed by the actor to external or situational factors, while the recipient attributes these outcomes to the actor’s inherent traits. In the context of parent–child interactions, when parents engage in phubbing, they might perceive their smartphone use as occasional and driven by external factors like work requirements. Conversely, children may interpret their parents’ phubbing as a habitual and consistent trait. This divergence in attribution leads to subjective cognitive biases regarding the objective phenomenon of parental phubbing.

Moreover, empirical research suggests that parental phubbing, unlike overt negative parenting behaviors, possesses distinctive behavioral and affective traits that contribute to the attenuation of parental attention [40]. Firstly, overt negative parenting behaviors are typically observable, such as physical aggression or verbal abuse, making them readily acknowledged by parents and the public, with clear recognition of their potential developmental risks for children. In contrast, parental phubbing is subtle, inconspicuous and concealed [41,42]. For parents, discerning the adverse impact of phubbing on children can be challenging. Secondly, overt negative parenting behaviors, like rejection and aggression, are often accompanied by intense negative emotions like anger. This makes it easier for parents to recognize and reflect upon the hazards of these negative parenting practices. Conversely, parental phubbing usually occurs during periods of emotional stability rather than strong negative affect, contributing to parents’ limited awareness of the potential negative consequences of such behavior [43]. This fosters a perceptual bias wherein parents perceive their phubbing as less conspicuous than it may objectively be.

### 4.2. The Impact of Parent–Child Discrepancies in the Perception of Parental Phubbing on Adolescent Smartphone Dependence and the Mediating Role of Parent–Child Relationship

The empirical analyses, encompassing both the correlation study and the response surface analysis, distinctly reveal that differences in how parents and children perceive parental phubbing significantly and positively influence adolescent smartphone dependence. In essence, the more pronounced these perceptual disparities become within parent–child pairs, the deeper the extent of smartphone dependence among adolescents. Viewing this through the lens of the “Discrepancy-Maladaptive” hypothesis, the heightened perceptual gap between parents and children sheds light on a noticeably weakened communicative connection between them. This may be attributed to a strained parent–child relationship or heightened conflict between adolescents and their parents [25,44]. Our findings regarding mediating effects further clarify that these noticeable differences in parent–child perceptions of parental phubbing are significantly and inversely related to the quality of the parent–child relationship. In simpler terms, when children report that their parents engage in substantial phubbing behavior while parents perceive their own phubbing behavior as minimal, the parent–child relationship often tends to be less favorable. In such circumstances, adolescents are more susceptible to negative emotional states. Furthermore, it demonstrates that increased perceptual disparities between adolescents and parents correlate with heightened maladaptive outcomes in adolescents, including anxiety, mood disturbances, conduct problems and substance use [21,45,46,47,48]. For adolescents caught in contentious parent–child relationships and frequently besieged by negative emotions, smartphones become outlets for emotional relief and repositories for psychological solace. In line with this, as emphasized by the latter portion of our findings on mediating effects, adolescents characterized by strained parent–child relationships exhibit a greater severity of smartphone dependence. Concurrent research indicates that adolescents within families marked by weak cohesion and suboptimal parent–child relationships are more inclined to seek external validation, with smartphones and the internet emerging as favored conduits [49]. These outlets serve as emotional regulators [50], often leading to an excessive reliance on smartphones among adolescents. The mediating role of parent–child relationships underscores the pivotal influence of the family. Consequently, effective strategies for preventing and intervening in adolescent smartphone dependence should consistently take the family environment into account. A harmonious and positive family setting can effectively mitigate a range of adverse adolescent behaviors.

On the other hand, conspicuous disparities in perception between parents and children shed light on parents’ limited awareness of their own phubbing behavior, which may directly contribute to the phenomenon of adolescent smartphone dependency. De Los Reyes suggests that when parent–child reports diverge, with adolescents reporting higher levels of behaviors than their parents (particularly in negative domains), it might indicate parents’ lack of insight into crucial aspects of adolescents’ lives, rendering them more susceptible to maladjustment [44]. In this context, these disparities unveil parents’ lack of discernment regarding their involvement in phubbing behavior. Parents often fail to fully comprehend the gravity of the consequences triggered by phubbing, perpetuating their indifference and potentially exacerbating such behavior, leading to significant ramifications. Research consistently affirms that children require active parental attention and responsiveness across various domains [51,52,53]. However, within the realm of familial interactions, parents’ engagement in phubbing emerges as a detrimental factor that disrupts parental attentiveness [54,55]. For children, parental phubbing equates to social ostracism [56]. When parents neglect to address the needs and expectations of their children or spend inadequate time with them, children feel neglected and excluded by their parents [40,57]. Children perceive their parents as distant, indifferent and overbearing, possibly prompting avoidance attachment strategies as a defense mechanism against repeated disappointments [58,59]. In light of these circumstances, children are compelled to seek alternative avenues for emotional fulfillment. Without proper guidance on how to seek appropriate emotional support, the easy accessibility and availability of smartphones naturally become the preferred outlets for emotional fulfillment, ultimately leading to smartphone dependency.

### 4.3. Limitations and Implications

This study acknowledges two noteworthy limitations. Firstly, the research design employed is cross-sectional in nature, which precludes making causal inferences. Future investigations should consider experimental and longitudinal approaches to establish causal relationships. Additionally, qualitative research could be a valuable avenue to explore this topic further. Qualitative research can provide a more in-depth understanding and interpretation of diverse perspectives. Secondly, the focus of this study is on Chinese adolescents, which limits the generalizability of the findings. Future research should include samples from diverse cultural contexts to enhance the universality of the study’s conclusions.

Previous research based on the “Discrepancy-Maladaptive” hypothesis has primarily focused on the concept of “discrepancy” within parental child-rearing practices, with the manifestations of “maladaptive” often concentrated in areas such as behavior, emotional states, social adaptation and academic achievement [21]. This study significantly broadens the scope of the “Discrepancy-Maladaptive” hypothesis by elucidating the mechanisms through which perceptual discrepancies between parents and children influence addictive behaviors. Our study positions the concept of “discrepancy” within the context of parental phubbing within the family, thereby extending the inquiry into adverse developmental outcomes to include the realm of addictive behaviors.

## 5. Conclusions

This study aimed to explore the relationships among parental phubbing’s perceptual discrepancies, parent–child relationships and adolescent smartphone dependence. The key conclusions drawn from the study are as follows:

The research unequivocally establishes the presence of substantial perceptual discrepancies between parents and adolescents concerning parental phubbing.

The investigation reveals that perceptual discrepancies in parental phubbing significantly and positively predict adolescent smartphone dependence while concurrently exerting a negative influence on parent–child relationships. Additionally, parent–child relationships demonstrate a significant negative prediction of adolescents’ smartphone dependence.

Parent–child relationships emerge as a pivotal mediating factor between the perceptual discrepancies in parental phubbing and adolescent smartphone dependence.

## Figures and Tables

**Table 1:** Correlations and descriptive statistics among variables.

	*M* (*SD*)	1	2	3	4	5
1. Parents perceive their own phubbing	21.61 (6.16)	-				
2. Students perceive parental phubbing	24.14 (7.59)	0.42 **	-			
3. Perception discrepancies of parental phubbing	0 (1.07)	−0.05	0.15 **	−		
4. Parent–child relationship	3.48 (0.86)	−0.15 **	−0.41 **	−0.22 **	−	
5. Adolescent smartphone dependence	2.42 (0.88)	0.12 **	0.32 **	0.16 **	−0.25 **	-

Note: ** *p* < 0.01; abbreviations *M*: mean; *SD*: standard deviation.

**Table 2:** The coefficients for the polynomial regression model and response surface analysis.

	Regression Coefficient						Response Surface Analysis Coefficients			
	*b*_0_ (*SE*)	*b*_1_ (*SE*)	*b*_2_ (*SE*)	*b*_3_ (*SE*)	*b*_4_ (*SE*)	*b*_5_ (*SE*)	*a*_1_ (*SE*)	*a*_2_ (*SE*)	*a*_3_ (*SE*)	*a*_4_ (*SE*)
Adolescent smartphone dependence										
Parental phubbing	2.56 *** (0.05)	0.17 *** (−0.3)	−0.01 (0.03)	−0.03 (0.02)	−0.07 * (0.03)	−0.01 (0.03)	0.15 *** (0.05)	−0.09 (0.03)	0.18 * (0.07)	0.05 (0.06)

Note: * *p* < 0.05, *** *p* < 0.001.

**Table 3:** Regression analysis of the mediating role of parent–child relationship.

Predictor	Criterion: Parent–Child Relationship				Criterion: Adolescent Smartphone Dependence			
	*β*	*SE*	*t*	[LLCI, ULCI]	*β*	*SE*	*t*	[LLCI, ULCI]
Grade	−0.14	1.13	−3.95 **	[−6.68, −2.29]	−0.06	2.06	−1.53	[−7.24, 0.85]
Perception discrepancies of parental phubbing	−0.2	0.53	−5.36 ***	[−3.85, −1.80]	0.12	0.97	3.27 ***	[1.25, 5.06]
Parent–child relationship					−0.23	0.07	−6.21 ***	[−0.56, −0.29]
*R* ^2^	0.07				0.08			
*F*	27.08 ***				19.83 ***			

Note: ** *p* < 0.01, *** *p* < 0.001; grade: the first grade of junior high school = 1, second year of junior high school = 2.

## Data Availability

The data presented in this study are available on request from the corresponding author. The data are not publicly available due to privacy.
